# Dengue virus serological prevalence and seroconversion rates in children and adults in Medellin, Colombia: implications for vaccine introduction

**DOI:** 10.1016/j.ijid.2017.02.016

**Published:** 2017-05

**Authors:** Mabel Carabali, Jacqueline Kyungah Lim, Diana Carolina Velez, Andrea Trujillo, Jorge Egurrola, Kang Sung Lee, Jay S. Kaufman, Luiz Jacinto DaSilva, Ivan Dario Velez, Jorge E. Osorio

**Affiliations:** a Dengue Vaccine Initiative, International Vaccine Institute, SNU Research Park, 1 Gwanak-ro, Gwanak-gu, Seoul, 151-742, South Korea; b Department of Epidemiology, Biostatistics and Occupational Health, McGill University, Montreal, Quebec, Canada; c Program for the Study and Control of Tropical Diseases, Universidad de Antioquia, Medellín, Colombia; d Department of Pathobiological Sciences, University of Wisconsin, Madison, Wisconsin, USA

**Keywords:** Dengue, Serological survey, Dengue prevalence, Dengue seroconversion, Dengue burden, Colombia

## Abstract

**Background:**

Dengue is an important public health problem worldwide. A vaccine has recently been licensed in some countries of Latin America and Asia. Recommendations for dengue vaccine introduction include endemicity and a high serological prevalence of dengue in the territories considering its introduction.

**Methods:**

A community-based survey was conducted to estimate dengue seroprevalence and age-specific seroconversion rates in a community in Medellin, Colombia, using a dengue serological test (IgG indirect ELISA). Residents were selected at random and were first screened for dengue infection; they were then followed over 2.5 years.

**Results:**

A total of 3684 individuals aged between 1 and 65 years participated in at least one survey. The overall dengue seroprevalence was 61%, and only 3.3% of seropositive subjects self-reported a past history of dengue. Among dengue virus (DENV)-naïve subjects with more than two visits (*n* = 1002), the overall seroconversion rate was 8.7% (95% confidence interval 7.3–10.4) per 1000 person-months, over the study period. Overall, the mean age of DENV prevalent subjects was significantly higher than the mean age of seroconverted subjects. Specifically, DENV seropositivity over 70% was observed in participants over 21 years old. Serotype-specific plaque-reduction neutralization tests (PRNT) revealed that all four dengue serotypes were circulating, with DENV4 being most prevalent.

**Conclusions:**

These laboratory-based findings could inform dengue vaccine decisions, as they provide age-specific seroprevalence and seroconversion data, evidencing permanent and ongoing dengue transmission in the study area. This study provides evidence for the existing rates of secondary and heterotypic responses, presenting a challenge that must be addressed adequately by the new vaccine candidates.

## Highlights

• A total of 3684 individuals between 1 and 65 years old participated in the survey.
• The overall seroprevalence by IgG Indirect ELISA was 61%.
• The overall seroconversion rate was 8.7% per 1000 person-months over 2.5 years.
• The highest rate of infection was observed during the fifth visit (February 2014).

## Graphical abstract

**Figure:**
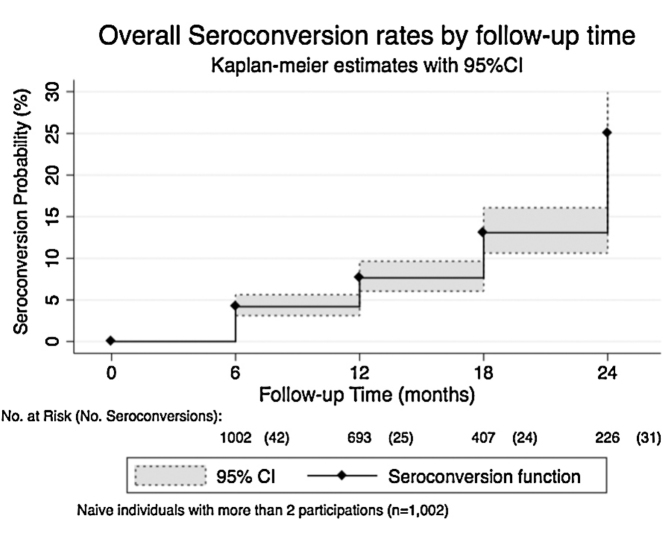

## Introduction

Dengue is considered a major cause of morbidity and mortality in tropical and sub-tropical countries.^1^, ^2^, ^3^, ^4^ It was estimated that in 2015, 79.6 million dengue cases occurred in 196 countries.^5^ Dengue is caused by any of the four antigenically distinct dengue virus serotypes (DENV1, DENV2, DENV3, and DENV4) and is transmitted to humans by the Aedes mosquitoes, the same vectors as for yellow fever virus, chikungunya virus, and Zika virus.^1^, ^6^, ^7^, ^8^, ^9^

Dengue infections range from inapparent or mild forms (with or without warning signs), to severe forms that can eventually lead to a fatal outcome.^1^, ^4^, ^7^, ^8^, ^9^, ^10^ The presence of warning signs and severe forms is often associated with secondary or subsequent infections by a heterotypic serotype, and may be caused by an immune phenomenon called antibody-dependent enhancement (ADE).^4^, ^6^, ^8^, ^9^, ^10^ Individuals infected by any of the DENV serotypes develop protective monotypic immunity evidenced by the generation of dengue immunoglobulin M (IgM) and immunoglobulin G (IgG) antibodies. The IgG antibodies may be found in serum at the end of the convalescent period (9–10 days) in primary infections, and may also be detected earlier in the case of secondary infections. IgG is found at higher titers up to 30–40 days after infection, but may remain detectable for decades, which allows the identification of individuals who have been in contact with the virus.^4^, ^9^, ^10^, ^11^

In Colombia, there is active circulation of all four serotypes. Epidemic waves have been occurring every 3–4 years, with shorter inter-epidemic periods, and the incidence rate is up to 220 cases per 100 000 inhabitants per year.^12^, ^13^, ^14^, ^15^ Although there is an existing surveillance system in Colombia to collect and provide information about symptomatic cases, there is limited information on inapparent infection or mild cases of dengue in people who do not seek medical care.^12^, ^15^, ^16^

A dengue vaccine has recently been licensed in some countries of Latin America and Asia, while there are other candidates in the pipeline.^17^, ^18^ Since both prevalence and transmission intensity are important factors that affect the efficacy and effectiveness of dengue vaccines,^17^, ^18^, ^19^, ^20^, ^21^, ^22^ it is important to acquire further knowledge about the burden of dengue in potential early dengue vaccine introducer countries such as Colombia.^19^ In order to better inform dengue vaccine introduction strategies in Colombia, the dengue serological prevalence and the seroconversion rate of individuals between 1 and 65 years of age in Medellin was estimated using a tiered dengue serological survey approach. The details and results of this study are described herein.

## Methods

### Study site and population

Medellin is the second largest city in Colombia with more than 2.6 million inhabitants. The reported annual incidence of dengue over the last 10 years has ranged between 161 and 745 cases per 100 000 inhabitants. Santa Cruz (Comuna 2) is one of the 16 sub-districts of Medellin and is located in the northeastern part of the city. The population in 2011, distributed across 12 neighborhoods, was 108 706 inhabitants; 57.6% of these inhabitants were female and 80% were aged under 65 years. Approximately 87% of the population in the community is Mestizo or white and 12% are Afro-Colombian. Approximately 96% of households belong to a low socioeconomic stratum and the remaining 4% belong to the ‘low–low’ stratum.^23^

### Study design

A tiered community-based serological survey was conducted in healthy residents of the 12 neighborhoods of Santa Cruz Comuna, from November 2011 to February 2014. To prevent any under-representation, the age and sex distributions of the Santa Cruz population, as well as the reported number of dengue cases in Medellin, were used to calculate sample sizes for age-stratified serum collection; these included the 95% confidence intervals and a margin of error at a fixed significance level within 25% of the true proportion of incidence, giving a relative precision of 75%, considering the gap in evidence for dengue incidence in the study areas. The sample size for precision is equal to^24^:

$$n=\frac{\left\{z^{2}\left[p\left(1-p\right)\right]\right\}}{{\left(p\times 0.25\right)}^{2}}$$

where *z* = 1.96 (i.e., the *z*-score for the desired 95% confidence interval) and *p* is the anticipated population (prevalence according to surveillance data). Using the formula above, the sample size for the serological survey was calculated using the adjusted incidence estimates, ranging from 0.116 to 0.312 depending on the age group.^14^ The age distribution was obtained from census data,^23^ and the age-specific sample sizes were calculated by adding what was obtained for each age group and assuming a non-response rate or loss to follow-up of about 10%. The total sample size required for individuals between 1 and 65 years of age was 2000 subjects per visit, to be followed up every 6 months during the study period.

Study outcomes were DENV seroprevalence (i.e., presence of DENV-specific antibodies in serum) and seroconversion (i.e., change from negative DENV antibodies during the first visit to positive DENV antibodies in subsequent visits), both assessed using IgG ELISA.

All residents of the district aged between 1 and 65 years were eligible. Individuals from the same household and participants presenting fever at the time of the survey could be enrolled. However, individuals participating in any dengue vaccine trial or with plans to move out of the catchment area during the following 6 months were not eligible.

### Data collection

Participants were located in their households; each household was selected using probabilistic multistage sampling, first randomly selecting the blocks and then selecting the households systematically. If the subjects were considered eligible for enrollment, the study staff described the study, invited them to participate, obtained informed consent, and then proceeded to collect a sample. Arrangements for the second visit, about 6 months after the first visit, were made before leaving the household. The same procedures were followed to take successive blood samples at subsequent visits; laboratory results were provided and explained at these subsequent visits.

Data were collected on the case record form (CRF) and then double-entered into a Microsoft FoxPro database. CRFs included the subject’s demographic data, medical history (including comorbidities and self-reported previous dengue infection), relevant symptoms (including the presence of fever, headache, muscle pain, and arthralgia, which were then asked about from the second visit onwards), and laboratory data. Financial compensation for lost wages was provided to the participants of the serological survey at each visit (maximum of 6 USD or its equivalent in Colombian Pesos (COP) in a voucher per person).

### Sample collection and antibody detection

Blood samples (7–10 ml) were obtained by trained phlebotomists. The specimens were scanned and logged into a computerized database. Whole blood was centrifuged and serum was separated into cryotubes under sterile conditions; 0.2–0.5 ml serum aliquots were labeled and stored at −80 °C until processing. All samples collected were tested with the Panbio Dengue IgG indirect ELISA.

### Plaque reduction neutralization test (PRNT)

The PRNT_50_ was conducted for samples indicating seroconversion or inconclusive results by IgG indirect ELISA. The PRNT analyses were based on previously described protocols.^20^ Virus strains used for the PRNT were DENV1 16007, DENV2 16681, DENV3 16562, and DENV4 1036. The reaction was revealed with AEC (3-amino-9-ethylcarbazole) chromogen and the immunofoci were counted using an ELISpot reader (AID Instruments, Germany). The PRNT titer was obtained from the reciprocal of the dilution of serum that reduced the plaque number by at least 50% relative to the virus-only control. Serum dilution values were expressed as the reciprocal of the original serum dilution, including the 1:2 dilution factor introduced during the neutralization step. Positive PRNT samples were defined as having a neutralizing titer of >1:10 to any of the viruses tested.

Samples that were first negative but then positive to any DENV serotype in subsequent samples by PRNT were considered as primary infection. Samples with previous neutralizing antibodies and positive to at least two or more DENV serotypes in subsequent samples were defined as secondary infection. Non-reactive cases were determined by the absence of neutralizing antibodies in both samples. Non-recent infections were defined when neutralizing antibodies were present in both samples and no increases or reductions in the PRNT_50_ titer were observed over time. Monotypic and heterotypic immune responses were defined as positive responses against single or multiple serotypes, respectively.

### Ethics

This study obtained ethical approval from the Ethics Committee of the University of Antioquia, Municipal Health Office (Secretaria de Salud de Medellin) and the Institutional Review Board of the International Vaccine Institute (IVI). Written informed consent was obtained from adult participants and informed consent was obtained from the parents or legal guardians of minors (including the assent of children between 7 and 17 years of age), by a signature or thumbprint prior to blood collection.

### Statistical analysis

Descriptive methods were used to present the general characteristics of all subjects. Summary statistics are presented as the mean with standard deviation (SD), frequency, or proportion depending on the variable. A mean comparison test was performed to estimate the difference in age as a continuous variable. To determine other statistical differences in the distribution of other key variables, the Kruskal–Wallis equality-of-populations rank test was performed.

Overall seroprevalence was calculated among subjects participating in at least one survey, and visit-specific seroprevalence rates were calculated using the total number of participants per visit as the denominator. Univariate and multivariate logistic regression was conducted to estimate the odds ratio (OR) with its corresponding 95% confidence interval (95% CI). In addition, multivariate logistic regression using a fixed-effects model adjusted by visits was performed to determine the characteristics related to DENV seroprevalence or seroconversion as the dependent variable. Independent variables in all models included age group, sex, ethnicity, neighborhood, and self-reported variables including previous condition/comorbidities, previous dengue, and yellow fever immunization (YFI) status. For the seroconversion model, presence of fever or other constitutional symptoms and ‘sought care’ during the 6 months prior to the visit were also added. Both models included an interaction term for age and sex, and another for age and previous conditions.

Person-months of follow-up time were calculated as the time between the date of the first visit and (1) the visit when seroconversion was identified, and (2) the last date of participation in the study, right-censoring. Age-specific seroconversion rates were calculated in naïve subjects (i.e., subjects with negative IgG indirect ELISA at their first visit) using the proportional follow-up person-months and reported as the age-specific seroconversion incidence rate per 1000 person-months with its corresponding 95% CI.

A *p*-value lower than 0.05 (*p <* 0.05) was considered as statistically significant. There were no imputations of missing data; therefore, denominators vary by response. The data analysis was conducted using Stata 13 (StataCorp LP, College Station, TX, USA). Maps were created using QGIS 2.14-Essen (http://www.qgis.org/).

## Results

### Enrolment strategy and characteristics of enrollees

**Figure 1:**
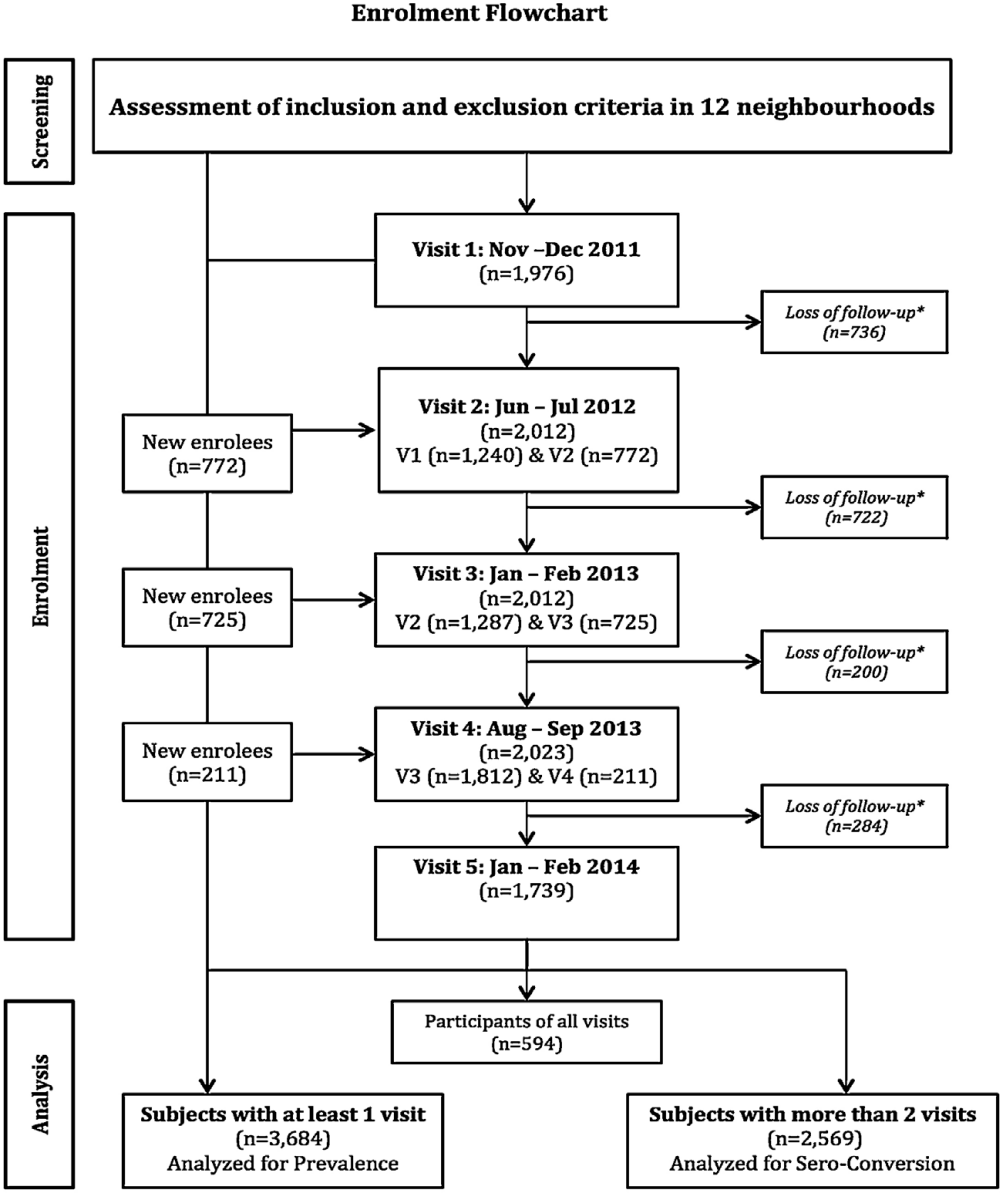
Flow chart of enrollment and participation.

Overall, 3684 individuals participated in at least one survey (Figure 1); 2450 (66.5%) were female, the mean age of all participants was 24.6 years (SD 17.1), and 3596 (97.6%) were Mestizo or white (Table 1). The median follow-up time was 12 months (interquartile range 0–18 months), with 2569 subjects participating in more than two surveys. Of those participants, 39% (*n* = 1002) were DENV-naïve subjects.

### Seroprevalence

**Figure 2:**
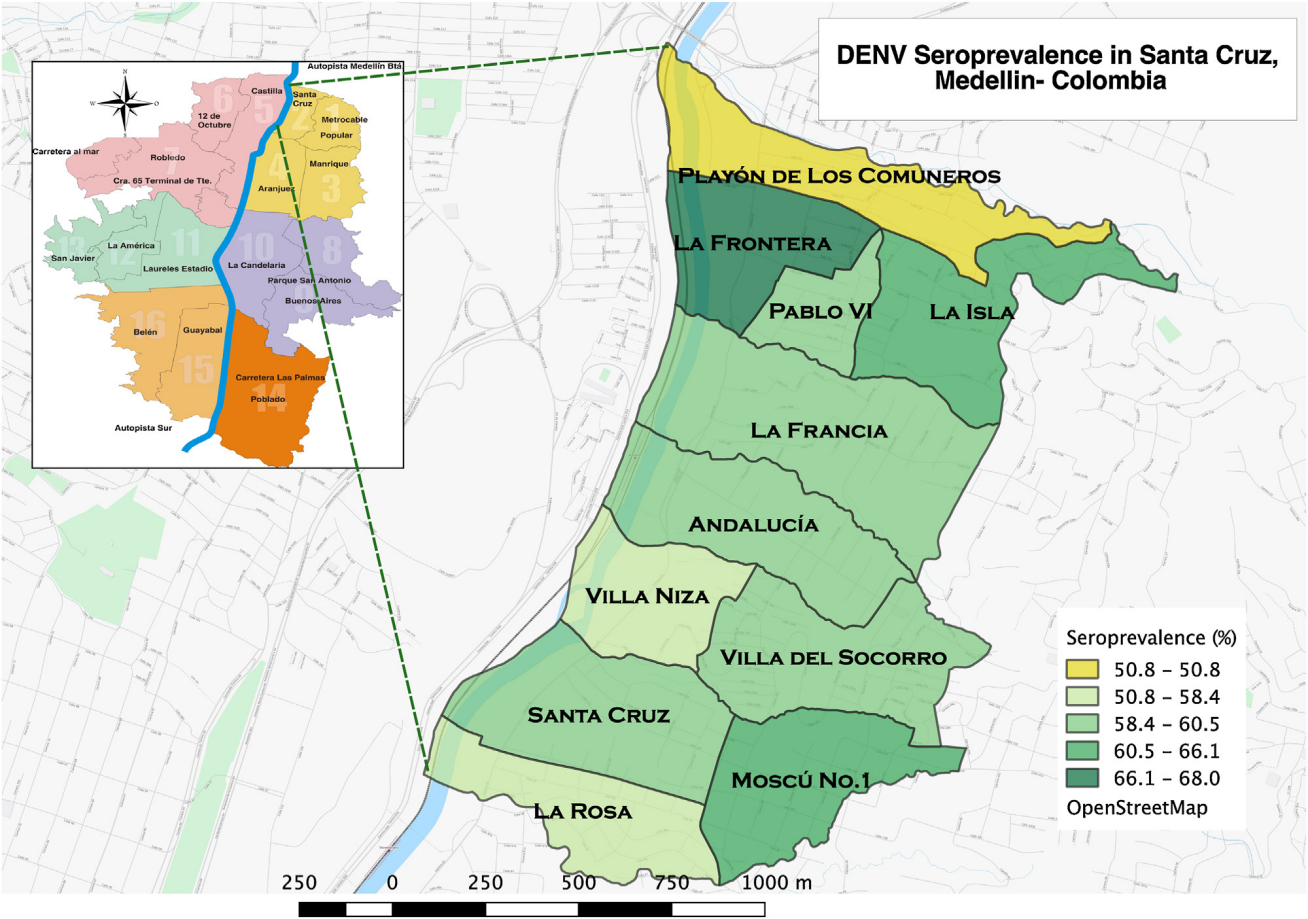
Overall dengue virus (DENV) seroprevalence by neighborhood. Map of Santa Cruz sub-district, indicating the proportion of DENV serological prevalence by IgG indirect ELISA in each neighborhood.

The overall DENV-positive serological prevalence by indirect IgG ELISA was 61% (*n* = 2246/3684; 95% CI 59.4–62.5%), with 3.2% (*n* = 73/2246) of seropositive subjects self-reporting a past history of dengue. The seroprevalence rates were as follows: first visit 59.8% (1182/1976), second visit 62% (1247/2012), third visit 62.6% (1259/2012), fourth visit 61.7% (1249/2023), and fifth visit 61.3% (1739/1066). The DENV prevalence status for the new enrollees was not significantly different (Chi-square = 8.1, df = 4, *p* = 0.087; Kruskal–Wallis test). DENV serological prevalence by neighborhood is presented in Figure 2.

The mean age of subjects with DENV antibodies was 30.3 years (SD 16.3) and without DENV antibodies was 15.6 years (SD 14.1); the difference between these groups was statistically significant (difference 14.7, 95% CI 13.7–15.7; *p <* 0.001). After controlling for visits and adjusting for all other covariates, there was a significant increase in the odds of DENV prevalence in subjects over 6 years old, especially in subjects between 41 and 50 years old (OR 47, 95% CI 36.1–61.1), when compared to subjects in the 1–5 years age group. There was also a significant increase in the odds of DENV prevalence in subjects with self-reported previous dengue infection (OR 4.7, 95% CI 3.0–7.4) compared to those who did not report previous dengue infection (Table 2). After including an interaction term between sex and age, it was observed that the odds of DENV prevalence increased when comparing males at 11–15 years to the reference group (OR 4.0, 95% CI 3.0–5.4; *p <* 0.001), and when comparing males to females at 16–20 years (OR 1.4, 95% CI 1.0–1.8; *p* = 0.043). However, the odds of seroprevalence decreased when comparing males to females at 11–15 years (OR 0.7, 95% CI 0.6–0.8; *p <* 0.001). Other interactions for age and sex were not statistically significant (data not shown).

### Seroconversion

**Figure 3:**
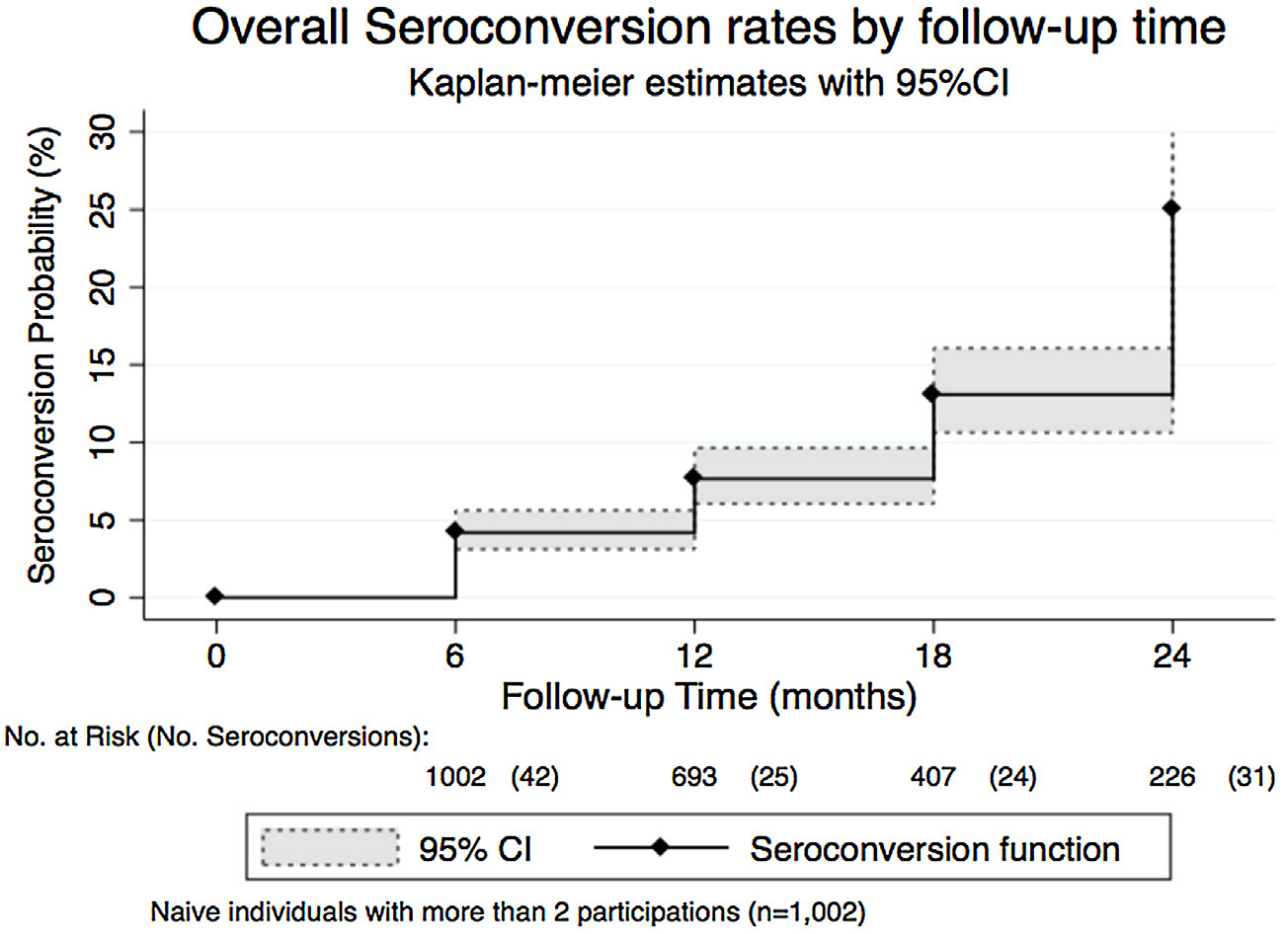
Seroconversion rate per visit. Overall seroconversion rate in dengue-naïve subjects at baseline by follow-up time. Includes the number at risk at the beginning of the time period and the number of events (new infections) in parenthesis. The curve shows an increasing probability of seroconversion over time.

**Figure 4:**
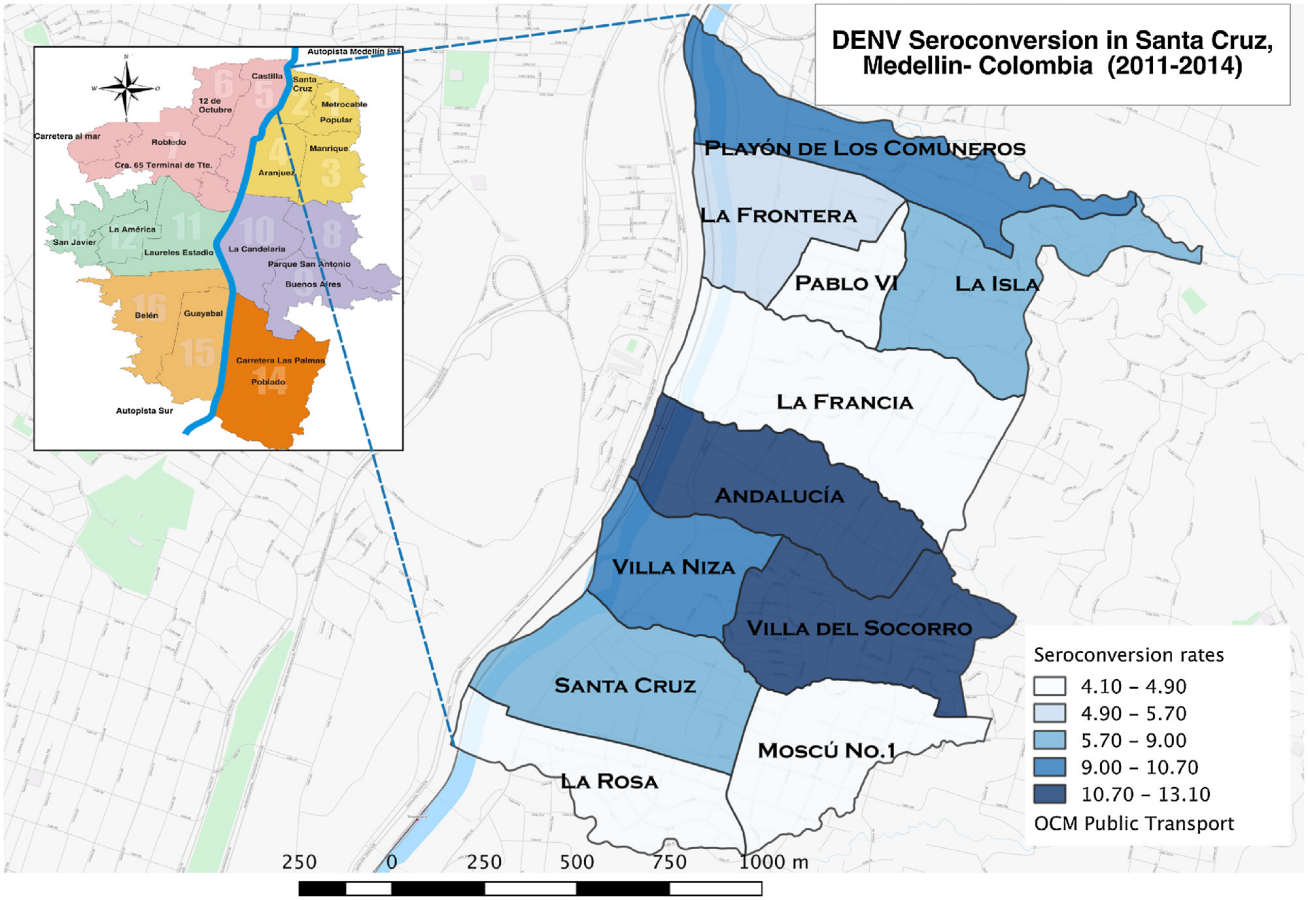
Distribution of seroconversion by neighborhood (rate). Map of Santa Cruz sub-district indicating the proportion of dengue virus seroconversion (negative to positive antibodies) by IgG Indirect ELISA in each of the 11 neighborhoods.

There were 122 new infections among the naïve subjects participating in more than one visit (*n* = 1002) and the mean age of seroconverted subjects was 21.8 years (SD 16.2). The overall seroconversion rate was 8.7% per 1000 person-months (95% CI 7.3–10.4) over 2.5 years. However, infections were occurring at different rates; the highest rate was 22.9% per 1000 person-months, observed during the fifth visit (February 2014) (Figure 3). The highest age-specific seroconversion rate was 17.9% per 1000 person-months (95% CI 10.6–30.3), observed in subjects between 31 and 40 years of age (Table 3). Overall the mean age of DENV prevalent subjects was significantly higher than the mean age of seroconverted subjects (difference 8.4, 95% CI 5.5–11.5; *p <* 0.001). The seroconversion rates in the various neighborhoods are presented in Figure 4.

**Figure 5:**
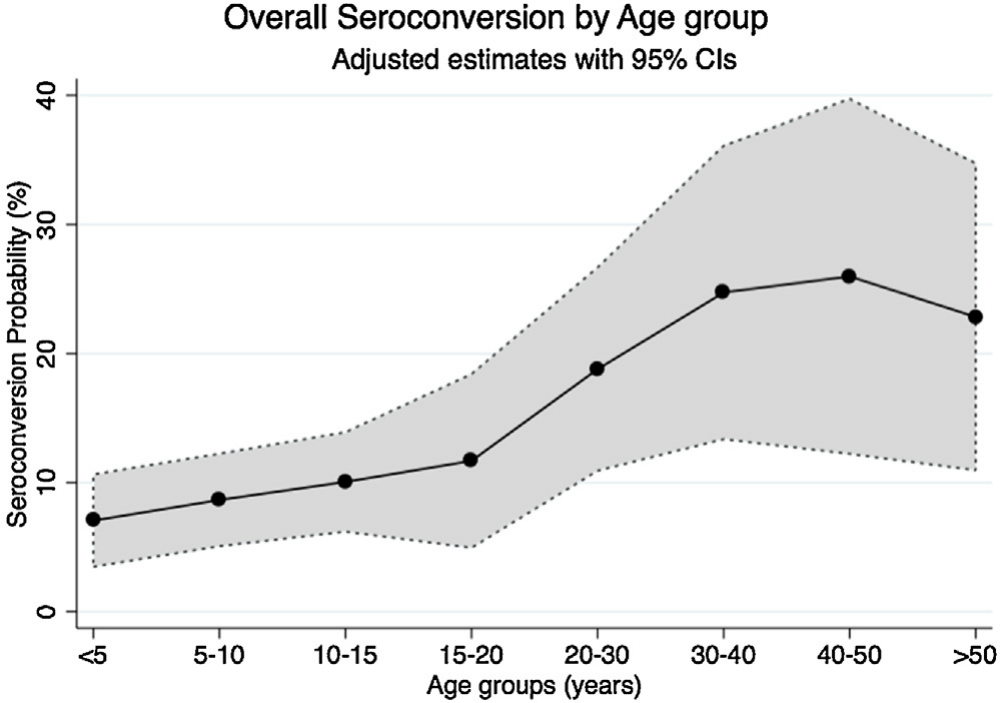
Overall probability of dengue seroconversion by age group. Seroconversion probabilities adjusted by sex, neighborhood, visits, presence of fever, and all other covariates in the model (*n* = 1002).

In both the univariate and multivariate analyses, age increased the odds of seroconversion, specifically in subjects between 31 and 40 years old (OR 3.8, 95% CI 1.7–8.6) when compared to subjects between 1 and 5 years old. After adjusting for all other variables, the odds of seroconversion were higher in males (OR 1.31, 95% CI 1.0–1.7), likely due to an interaction with age, and in Afro-Colombians (OR 3.0, 95% CI 1.2–7.4) (Table 4 and Figure 5).

### PRNT results

A suitable paired sample was not available for 10 of the seroconverted samples; analyses were conducted using only the remaining 112 samples. Overall, 93% (*n* = 104/112) of samples considered positive for DENV by IgG ELISA were found to be reactive by PRNT. Primary infection with monotypic and heterotypic responses was detected in 2.9% (3/104) and 3.8% (4/104) of samples, respectively. Secondary infection with monotypic and heterotypic responses was found in 3.8% (4/104) and 55.8% (58/104) of samples, respectively. There were 35 samples with neutralizing antibodies in which the PRNT_50_ did not show significant changes. The circulation of all four DENV serotypes in the study area was confirmed by PRNT identification. Predominant DENV serotypes were DENV1 (*n* = 36) and DENV4 (*n* = 26), followed by DENV2 (*n* = 20) and DENV3 (*n* = 14).

## Discussion

Dengue is a major public health issue in Colombia. According to the present study findings, there is significant evidence of an important dengue burden in Medellin, with an overall dengue serological prevalence as high as 61% among residents of Santa Cruz aged 1–65 years, and an overall seroconversion rate of 8.7% per 1000 person-months during the study period. Despite this important prevalence, community self-awareness of dengue infection is low − only 3.3% of prevalent subjects self-reported at least one dengue episode. Although similar findings have been reported in other Latin American settings,^25^, ^26^ a change in people’s awareness is foreseeable due to the increasing public health strategies in place to diagnose and control the infections transmitted by Aedes mosquitoes, specifically for DENV and Zika virus.

The increasing odds of seroprevalence in the older age groups could be attributed to the age effect in terms of time as opportunity of exposure: older people have had more time to be exposed than young people. It could also indicate that in the study population, individuals were exposed to dengue at an older age rather than during the earlier years of life. This could also be attributed to the fact that the study was conducted after one of the biggest outbreaks to have occurred in Colombia (2010), probably increasing the odds of exposure to DENV of susceptible adults before the beginning of this study, increasing the overall seroprevalence and depleting susceptible people.^14^, ^15^, ^16^, ^27^, ^28^ This aspect is important at the time of dengue vaccine introduction.^18^, ^19^, ^28^, ^29^, ^30^ Although a new dengue vaccine has been licensed for use in individuals between 9 and 45 years of age or between 9 and 60 years of age, the recommendation for introduction according to the license includes endemic settings with around 70% or greater DENV serological prevalence in the group targeted for dengue immunization.^17^

Interestingly, in terms of frequencies, the majority of seroconversions were in subjects under 15 years of age; however, rates were higher among adults with lower seroconversion rates at the extremes. These findings are comparable to observations made in Puerto Rico and Nicaragua,^25^, ^31^ where dengue incidence seems to increase with age, a pattern that is common in Latin American countries but which contrasts with the pattern observed in Southeast Asia.^16^, ^28^, ^32^, ^33^ Although it was outside the scope of this study, the assessment of neighborhood-specific strategies for vector control and other environmental characteristics might have been helpful for understanding the relatively homogeneous distribution of DENV prevalence and the more variable behavior for seroconversion. In Latin America, risk assessment by environmental (including entomological evaluation), socioeconomic, and health care access characteristics has been considered a useful tool to identify the dengue burden.^15^, ^25^, ^34^, ^35^ Moreover, comparing neighborhoods or other small geographic units to identify areas with dengue is key to address vector control and other targeted strategies for disease control.^11^, ^15^, ^19^, ^25^, ^31^

In this study there was no significant difference between the sexes or ethnic groups in DENV prevalence, as described previously in other settings.^4^, ^16^, ^26^, ^32^, ^36^ However, the seroconversion rate seemed to be increased in Afro-Colombians compared to whites. Although an increase in seroconversion rate was expected in all ethnic groups because a new outbreak was starting at the time of the fourth visit (2013), it will be important to look carefully at the distribution of DENV-susceptible individuals and the seroconversion in the study area, where the majority of the population is Mestizo/white and for which an increased risk of infection and severity have been described.^11^, ^16^, ^26^, ^27^, ^36^

In the Colombian context, YFI is recommended but not compulsory for people travelling to endemic areas.^37^ A relatively small population reported having been immunized against yellow fever (28.8%). After adjusting for all covariates, prior YFI was not associated with seropositivity but was associated with seroconversion. These findings together with the possibility of cross-reactivity should be interpreted with caution and could be compared to what has been reported in other studies performed in Central America and Southeast Asia.^4^, ^16^, ^33^

Neither fever nor seeking care for fever during the 6 months prior to the visits was associated with seroconversion, although the presence of symptoms was. This is important for the assessment and definition of ‘asymptomatic’ dengue infection.^7^, ^15^, ^16^, ^22^, ^31^, ^38^ In this case, the incidence rate of dengue infections that were either mild (because the subject acknowledged the presence of symptoms) or inapparent is indicated, because only 9.8% of seroconverted subjects reported fever during the previous 6 months. Although similar rates have been observed in Nicaragua and in other endemic countries,^16^, ^27^, ^31^, ^33^ the rate of ‘subclinical’ dengue infection remains unclear, and in the presence of a low confirmation rate of dengue cases, the accurate identification of dengue burden remains challenging.^16^, ^33^, ^35^

The four DENV serotypes were identified by PRNT in the study area, which is associated with the observed high seroprevalence and indicates ongoing DENV transmission. The predominance of DENV4 and the low presence of DENV3 in the study area could also explain the presence of mild symptoms in seroconverted subjects.^4^, ^27^, ^32^, ^39^ This distribution of serotypes could also be related to the important circulation of DENV3 and DENV1 from the years 2000 to 2011,^15^ and specifically *during the 2010’s outbreak*.^14^, ^15^, ^26^, ^30^

### Strengths and limitations

Despite the known important burden of dengue in Colombia, this study is the first to address serological prevalence and age-specific seroconversion rates in the study population, based on laboratory data. The study period lasted around 2.5 years, allowing incidence rates for new dengue infections to be calculated – information that is needed and has been broadly discussed.^15^, ^35^ Knowing the seasonality of dengue, sensitive information about the patterns of dengue distribution were captured for the study area. As in other studies, the level of attrition related to cohort studies constitutes a limitation that has been described previously.^25^ Part of the large attrition seen at the first and second visits was due to a municipal urbanization relocation plan in one of the neighborhoods (Sinai), which was a slum. Inhabitants of this neighborhood were relocated not only within the study area but also elsewhere, and it was not possible to follow them up. This right-censoring was considered non-informative, and the absence of statistical difference in seroprevalence status in new enrollees eased concerns about the risk of a selection bias. The interpretation of self-reported previous dengue infection and presence of fever or other symptoms may have been affected by the known lack of awareness of the disease in some Latin American contexts,^15^, ^32^, ^38^ as well as the risk of recall bias because of the visit intervals.

Although it is important to acknowledge the possible presence of other arboviruses in the study area, this study was conducted prior to the documented introduction of chikungunya and Zika viruses, favoring the reliability of the serological analysis. However, the possibility that some secondary infections were missed by using the IgG ELISA to test for seroconversion and that some of the age effect observed in the seroprevalence may have been related to the intrinsic characteristics of the test is acknowledged. The PRNT was necessary to identify recent and non-recent heterogeneous infections in the presence of a high serological prevalence and the use of an IgG ELISA; however, the presence of heterotypic responses might limit the interpretations about the serotype-specific incident cases.

### Conclusions

The role of seroprevalence has been discussed largely in regard to the efficacy and the effectiveness of dengue vaccines.^18^, ^20^, ^21^, ^22^, ^28^, ^29^, ^30^, ^40^ From the public health point of view, it is important to have an estimate of age-specific dengue prevalence in endemic countries at the time of dengue vaccine introduction, as suggested by the World Health Organization.^11^, ^29^, ^30^ The information provided in this article has a two-fold impact. First, the age-specific seroprevalence and seroconversion rates will be helpful for policymakers, allowing informed decisions to be made on whether they should consider the introduction of dengue vaccines.^11^, ^12^, ^19^, ^22^, ^30^, ^41^ Specifically, due to the important serological prevalence, the evidence of ongoing transmission, the current re-emergence of other arboviruses, and other demographic characteristics of the study population, the introduction of dengue vaccine, along with a structured vector control program, could be considered when formulating disease control strategies. Secondly, for the developers of new dengue vaccines currently in the pipeline, this study provides evidence of the relative heterogeneity that can be observed in endemic areas such as Colombia and also shows the existing heterotypic response rates, which present a challenge that must be addressed adequately by the new vaccine candidates.^17^, ^18^, ^19^, ^21^, ^22^, ^28^, ^29^, ^30^, ^33^, ^40^, ^41^, ^42^ Overall, this study contributes to a better understanding of dengue in Medellin, Colombia, and in other Latin American settings with similar characteristics (i.e., low socioeconomic urban and peri-urban areas with intermediate to high prevalence and areas endemic for dengue).

## Figures and Tables

**Table 1:** General characteristics of study subjects and DENV prevalence at enrolment

Characteristics	Total (*N* = 3684)^a^	DENV seropositive (*n* = 2246)^b^
	*n* (%)	*n* (%)
Sex, female	2450 (66.5)	1617 (72.0)
Ethnicity		
White (Hispanic)	109 (3.0)	67 (3.0)
Mestizo	3487 (94.6)	2134 (95.0)
African descendent	88 (2.4)	45 (2.0)
Age, mean ± SD	24.6 ± 17.1	30.3 ± 16.3^c^
Age groups, years		
1–5	376 (10.2)	50 (2.2)
6–10	487 (13.2)	145 (6.5)
11–15	552 (15.0)	279 (12.4)
16–20	405 (11)	276 (12.3)
21–30	615 (16.7)	457 (20.3)
31–40	429 (11.6)	341 (15.2)
41–50	414 (11.2)	362 (16.1)
51–60	298 (8.1)	243 (10.8)
>60	108 (2.9)	93 (4.1)
Neighborhood		
La Isla	254 (6.9)	168 (7.5)
Villa del Socorro	309 (8.4)	157 (7.0)
El Playon de los Comuneros	296 (8.0)	178 (7.9)
Villa Niza	356 (9.7)	243 (10.8)
Pablo VI	275 (7.5)	163 (7.3)
Moscu 1	311 (8.4)	189 (8.4)
La Frontera	226 (6.1)	134 (6.0)
Santa Cruz	399 (10.8)	233 (10.4)
La Francia	344 (9.3)	228 (10.1)
La Rosa	368 (10.0)	222 (9.9)
Andalucía	274 (7.4)	158 (7.0)
Sinai	272 (7.4)	173 (7.7)
Pre-existing conditions^d^	721 (19. 6)	491 (21.9)
Self-reported previous dengue infection	84 (2.3)	73 (3.3)
Yellow fever vaccination^e^		
No	782 (21.2)	515 (22.9)
Yes	1061 (28.8)	573 (25.1)
Don’t know	1841 (50.0)	1158 (51.6)

DENV, dengue virus; SD, standard deviation.

a Subjects with at least one participation.

b DENV IgG indirect ELISA positive at visit 1.

c The mean age difference between subjects with and without positive DENV antibodies at enrolment was 14.7 years (95% confidence interval 13.7–15.7 years); *p* < 0.001.

d Self-reported comorbidities or previous conditions such as diabetes, hypertension, hypercholesterolemia, etc.

e Self-reported yellow fever immunization status.

**Table 2:** Univariate and multivariate logistic regression analysis for DENV prevalence (IgG Indirect ELISA positive antibodies) at enrollment.

Characteristics (*N* = 3684)	Univariate analysis			Multivariate analysis^a^		
	OR	95% CI	*p*-Value	aOR	95% CI	*p*-Value
Sex						
Female	Ref.	–	–	Ref.	–	–
Male	0.54	(0.47–0.62)	0.001	1.01	(0.91–1.13)	0.831
Ethnicity						
White (Hispanic)	Ref	–	–	Ref.	–	–
Mestizo	0.99	(0.67–1.46)	0.935	0.77	(0.58–1.04)	0.084
African descendent	0.66	(0.37–1.16)	0.146	0.77	(0.50–1.18)	0.224
Age, years						
1–5	Ref.	–	–	Ref.	–	–
6–10	2.76	(1.94–3.94)	<0.001	2.83	(2.28–3.51)	<0.001
11–15	6.66	(4.74–9.37)	<0.001	6.07	(4.94–7.46)	<0.001
16–20	13.95	(9.70–20.07)	<0.001	12.65	(10.1–15.85)	<0.001
21–30	18.86	(13.31–26.72)	<0.001	20.10	(16.08–25.13)	<0.001
31–40	25.26	(17.30–36.90)	<0.001	27.14	(21.2–34.76)	<0.001
41–50	45.39	(29.94–68.81)	<0.001	46.97	(36.09–61.12)	<0.001
51–60	28.81	(18.98–43.73)	<0.001	34.09	(26.11–44.52)	<0.001
>60	40.42	(21.72–5.24)	<0.001	32.24	(22.19–46.85)	<0.001
Neighborhood						
La Isla	Ref.	–	–	Ref.	–	–
Villa Socorro	0.53	(0.38–0.74)	<0.001	0.41	(0.32–0.53)	<0.001
El Playon	0.77	(0.54–1.09)	0.146	0.61	(0.47–0.78)	<0.001
Villa Niza	1.1	(0.78–1.55)	0.582	0.87	(0.68–1.13)	0.298
Pablo VI	0.75	(0.52–1.06)	0.103	0.71	(0.55–0.91)	0.008
Moscu 1	0.79	(0.56–1.12)	0.188	0.64	(0.49–0.82)	<0.001
La Frontera	0.75	(0.51–1.08)	0.121	0.77	(0.59–1.01)	0.057
Santa Cruz	0.72	(0.52–1.00)	0.048	0.53	(0.42–0.68)	<0.001
La Francia	1.01	(0.71–1.42)	0.972	0.96	(0.73–1.24)	0.734
La Rosa	0.78	(0.56–1.09)	0.141	0.58	(0.45–0.75)	<0.001
Andalucía	0.7	(0.49–0.99)	0.046	0.51	(0.39–0.66)	<0.001
Sinai	0.89	(0.63–1.28)	0.542	0.61	(0.47–0.80)	<0.001
Pre-existing conditions^b^	1.47	(1.24–1.75)	<0.001	0.97	(0.85–1.11)	0.678
Previous DENV^c^	4.36	(2.30–8.24)	<0.001	4.70	(2.98–7.41)	<0.001
Yellow fever vaccination^d^						
No	Ref.	–	–	Ref.	–	–
Yes	0.61	(0.50–0.74)	<0.001	1.08	(0.96–1.22)	0.215
Don’t know	0.88	(0.74–1.05)	0.150	1.05	(0.93–1.19)	0.447

DENV, dengue virus; OR, odds ratio; CI, confidence interval; aOR, adjusted odds ratio.

a Multilevel logistic regression by visit, using a random-effects model, indicating the aOR of DENV prevalence on each covariate.

b Self-reported comorbidities or previous conditions such as diabetes, hypertension, hypercholesterolemia, etc.

c Self-reported previous dengue infection.

d Self-reported yellow fever immunization status.

**Table 3:** Age-specific and sex distribution of seroconversion cases and the corresponding rates per 1000 person-months in naïve subjects (seronegative at visit 1) with more than one visit (*n* = 1002).

Age group, years	Seroconversion					
	Female, *n* (%)	Male, *n* (%)	Total	Person-months	Rate	95% CI
1–5	5 (6.7)	9 (19.1)	14	2916	4.8	(2.9–8.2)
6–10	8 (10.7)	13 (27.7)	21	3210	6.5	(4.2–10.0)
11–15	12 (16.0)	12 (25.5)	24	3192	7.5	(5.0–11.1)
16–20	7 (9.3)	3 (6.4)	10	1218	8.2	(4.4–15.3)
21–30	14 (18.7)	4 (8.5)	18	1260	14.3	(8.9–22.5)
31–40	12 (16.0)	2 (4.3)	14	780	17.9	(10.6–30.3)
41–50	8 (10.67)	2 (4.3	10	594	16.8	(9.1–31.3)
>50	9 (11.9)	2 (4.3)	11	798	13.8	(7.6–24.7)
Total	75 (100)	47 (100)	122	14 028	8.7	(7.3–10.4)

CI, confidence interval.

**Table 4:** Univariate and multivariate logistic regression for seroconversion in naïve subjects (seronegative at visit 1) with more than one visit (*n* = 1002).

Seroconverted subjects (*n* = 122/1002)		Univariate analysis			Multivariate analysis^a^		
Characteristics	*n* (%)	OR	95% CI	*p*-Value	aOR	(95% CI)	*p*-Value
Sex							
Male	47 (38.52)	0.84	(0.57–1.24)	0.379	1.31	(1.02–1.68)	0.036
Ethnicity							
White (Hispanic)	3 (2.46)	Ref.	–	–	Ref	–	–
Mestizo	113 (92.62)	1.04	(0.31–3.52)	0.950	1.06	(0.52–2.18)	0.866
African descendent	6 (4.92)	1.92	(0.43–8.58)	0.395	3.01	(1.22–7.42)	0.017
Age groups, years (mean 21.8, SD 16.2)							
1–5	14 (11.48)	Ref.	–	–	Ref.	–	–
6–10	21 (17.21)	1.3	(0.65–2.66)	0.445	1.43	(0.95–2.14)	0.087
11–15	24 (19.67)	1.66	(0.84–3.31)	0.148	1.67	(1.12–2.48)	0.011
16–20	10 (8.20)	1.76	(0.75–4.13)	0.195	1.60	(0.94–2.72)	0.085
21–30	18 (14.75)	3.09	(1.46–6.5)	0.003	4.17	(2.66–6.54)	<0.001
31–40	14 (11.48)	4.68	(2.08–10.57)	<0.001	4.73	(2.9–7.72)	<0.001
41–50	10 (8.20)	4.57	(1.86–11.22)	0.001	5.68	(3.33–9.7)	<0.001
51–60	10 (8.20)	4.29	(1.75–10.47)	0.001	5.36	(3.11–9.24)	<0.001
>60	1 (0.82)	1.37	(0.16–11.49)	0.771	1.69	(0.55–5.15)	0.357
Neighborhood							
La Isla	11 (9.02)	Ref.	–	–	Ref.	–	–
Villa Socorro	12 (9.84)	0.48	(0.20–1.16)	0.105	0.45	(0.27–0.76)	0.003
El Playon	6 (4.92)	0.34	(0.12–0.98)	0.046	0.32	(0.17–0.6)	<0.001
Villa Niza	7 (5.74)	0.46	(0.17–1.28)	0.138	0.39	(0.21–0.73)	0.003
Pablo VI	6 (4.92)	0.32	(0.11–0.93)	0.036	0.35	(0.19–0.65)	0.001
Moscu 1	16 (13.11)	1.0	(0.43–2.34)	0.995	1.02	(0.61–1.7)	0.95
La Frontera	14 (11.48)	1.05	(0.44–2.53)	0.908	1.45	(0.87–2.42)	0.152
Santa Cruz	13 (10.66)	0.56	(0.23–1.35)	0.195	0.47	(0.28–0.81)	0.007
La Francia	6 (4.92)	0.42	(0.15–1.22)	0.112	0.46	(0.24–0.88)	0.019
La Rosa	8 (6.56)	0.45	(0.17–1.19)	0.108	0.53	(0.29–0.94)	0.031
Andalucía	5 (4.10)	0.31	(0.10–0.95)	0.041	0.34	(0.18–0.65)	0.001
Sinai	18 (14.75)	1.31	(0.56–3.03)	0.53	1.24	(0.74–2.08)	0.411
Pre-existing condition^b^							
Yes	24 (19.67)	1.33	(0.82–2.15)	0.249	1.16	(0.86–1.55)	0.328
Previous DENV^c^							
Yes	4 (3.28)	4.94	(1.37–17.75)	0.014	4.12	(1.88–9.0)	<0.001
Yellow fever vaccination^d^							
No	27 (22.13)	Ref.	–	–	Ref.	–	–
Yes	53 (43.44)	1.08	(0.66–1.77)	0.749	1.41	(1.06–1.86)	0.017
Don’t know	42 (34.43)	1.82	(1.08–3.06)	0.023	1.78	(1.32–2.42)	<0.001
Fever^e^	12 (9.84)	0.97	(0.51–1.83)	0.924	1.18	(0.83–1.67)	0.367
Sought care^f^	6 (4.92)	1.42	(0.58–3.47)	0.446	1.56	(0.94–2.6)	0.084
Symptoms^g^	111 (90.98)	1.44	(0.75–2.76)	0.271	1.85	(1.16–2.94)	0.009

OR, odds ratio; CI, confidence interval; aOR, adjusted odds ratio.

a Multivariate logistic regression adjusted by visit, using a fixed-effects model, indicating the aOR of seroconversion on each covariate.

b Self-reported comorbidities or previous conditions such as diabetes, hypertension, hypercholesterolemia, etc.

c Self-reported previous dengue infection.

d Self-reported yellow fever immunization status.

e Self-reported presence of fever during the 6 months prior to the visit.

f Self-reported medical consultation due to the febrile episode.

g Self reported constitutional symptoms/ dengue-like symptoms (headache, myalgia, arthralgia, etc.)
